# Polysomnographic characteristics in patients with idiopathic dystonia

**DOI:** 10.1016/j.prdoa.2026.100427

**Published:** 2026-02-09

**Authors:** Lejla Paracka, Lan Ye, Jann Lübke, Assel Saryyeva, Martin Klietz, Florian Wegner, Joachim K. Krauss

**Affiliations:** a Department of Neurology, Hannover Medical School, Hannover, Germany; b Department of Neurosurgery, Hannover Medical School, Hannover, Germany

**Keywords:** Dystonia, Sleep, Polysomnography, Non-motor symptoms

## Abstract

**Background:**

Several aspects of the occurrence of sleep disturbances in dystonia remain unclear. Here, we assessed sleep architecture with polysomnography (PSG) and investigated the association of specific findings with mood, quality of life and severity of disease.

**Methods:**

Twelve patients with idiopathic dystonia underwent overnight PSG and findings were compared to those of a normative collective. Subjective sleep quality was evaluated with the Pittsburgh-Sleep-Quality-Index (PSQI) and the Epworth-Sleepiness-Scale (ESS). PSG findings, PSQI and ESS were correlated to motor scores of the Burke-Fahn-Marsden-Dystonia-Rating-Scale and the Torticollis-Western-Spasmodic-Rating-Scale. Further correlations were investigated between sleep assessment and mood (Beck-Depression-Inventory (BDI) and Hamilton-Depression-Rating-Scale (HDRS)), and quality of life (SF-36 subscales).

**Results:**

Patients with dystonia had impairments in REM sleep, increased N2 and arousal index. Impaired REM sleep was positively correlated with the SF-36 subscore mental health, the N1 sleep stage was associated with the HDRS score, the arousal index was negatively correlated with the SF-36 subscores social functioning and bodily pain, but positively correlated with the BDI, and daytime sleepiness according to ESS was correlated with the SF-36 subscale vitality. There were no significant correlations with motor scores.

**Conclusion:**

Our study demonstrates that sleep impairment is a fundamental characteristic in patients with idiopathic dystonia affecting both mood and quality of life. Sleep disturbances are not merely a secondary consequence of the motor disorder, but appear to be an integral and primary feature of dystonia itself.

## Highlights

• Idiopathic dystonia is associated with impaired sleep quality and architecture.
• Polysomnographic findings revealed impaired REM phase of sleep, increased arousal index and N2 Phase of sleep.
• Altered sleep quality adversely affect mood and quality of life in these patients.
• Sleep disturbance may constitute an intrinsic feature of the disease profile, independent of motor symptoms.

## 1 Introduction

Dystonia is a movement disorder characterized by sustained or intermittent abnormal movements, postures or both. Dystonic movements and postures are typically patterned, repetitive and may be tremulous and jerky. They are often initialized or worsened by voluntary action, and frequently associated with overflow movements [1]. Besides being known as a motor disorder, non-motor symptoms have been recognized as associated impairments in dystonia [2], [3], [4]. Sleep related issues in patients with idiopathic dystonia received relatively little attention in the past. Difficulties such as poor sleep quality, increased fatigue and excessive daytime sleepiness were reported as primary non-motor symptoms. The prevalence of patients with self-reported sleep impairment in focal dystonia ranges between 40% and 70% [5], [6]. These sleep complaints are related to impaired quality of life and depression symptoms [7], [8], [9], [10].

Sleep impairment in dystonia has been underinvestigated [11]. There are only few studies that have quantitatively evaluated sleep architecture in patients with dystonia by using polysomnography (PSG), the gold standard for sleep assessment. Among the limited number of investigations that compared polysomnographic findings between patients with dystonia and healthy control subjects, results have been partially inconsistent, and at times contradictory [12], [13], [14].

The aim of the present study was to comprehensively investigate the sleep architecture and the quality of sleep by using overnight polysomnography, along with questionnaires of self-reported sleep assessment, in a cohort of patients with idiopathic dystonia. We also aimed to examine the relationship between sleep disturbances, quality of life, mood, and severity of dystonia. Finally, we sought to explore whether subclinical sleep alterations – not necessarily meeting diagnostic thresholds – could exert a measurable influence on patients’ quality of life and mood, thereby revealing subtle but clinically relevant sleep-related impairments which might otherwise go undetected.

## 2 Materials and methods

Twelve patients (7 female, 5 male) with idiopathic dystonia (3 generalized, 6 segmental and 3 isolated cervical dystonia), scheduled for deep brain stimulation in Hannover Medical School were included in the study. Mean age at the time of admission was 53.9 years (±18.6). Disease duration was 13.8 years (±11.2). The Burke-Fahn-Marsden-Dystonia-Rating-Scale (BFMDRS) movement mean score was 17.7 points (±9.1), the Torticollis-Western-Spasmodic-Rating-Scale (TWSTRS) motor score was: for severity 10.8 points (±3.9); for disability 9.2 points (±4.5); pain 4.7 points (±3.2). All patients signed written informed consent prior to the study. The study was approved by the ethics committee of Hannover Medical School (No. 6307).

Patients with a diagnosis of idiopathic dystonia were considered eligible for inclusion in the study. Exclusion criteria were the use of medication known to influence sleep architecture, such as hypnotics, sedatives or antipsychotics. To avoid confounding effects, patients with comorbid sleep conditions were excluded, as these conditions could independently influence sleep architecture and obscure disease specific associations.

Additionally, patients with any acute medical illness at the time of the assessment were excluded from the study.

The assessment was performed at least 3 months after the last botulinum toxin therapy. Self-reported sleep was evaluated by the Pittsburgh-Sleep-Quality-Index (PSQI), where a score over 5 points on the global scale identified poor sleepers [15]. Daytime sleepiness was evaluated by the self-reported Epworth-Sleepiness-Scale (ESS), where scores above 9 are considered significant [16]. Mood was assessed with the self-reported Beck-Depression-Inventory (BDI) and the Hamilton-Depression-Rating-Scale (HDRS). BDI scores between 0 and 9 represent no depression, 10–18 mild depression, and 19–29 moderate to severe depression, while scores from 30 to 63 indicate severe depression [17]. On the HDRS, scores of 0–7 represent no depression, 8–16 mild depression, 17–23 moderate depression, and more than 24 severe depression [18]. The patients also completed the Short Form 36 (SF-36) health survey [19] to estimate health related quality of life. This scale is comprised of eight subscales: (1) physical functioning, (2) physical role functioning, (3) bodily pain, (4) general health perceptions, (5) vitality (energy and fatigue), (6) social functioning, (7) emotional role functioning and (8) mental health. The SF 36 scores (the higher the better) of patients with dystonia were compared to the SF 36 scores of 12 age matched healthy individuals [3].

All patients underwent one full night of PSG. The PSG data were acquired by using Somnolab 2 Software. Electrodes were placed on the scalp according to conventional EEG. Electrooculography and electromyography (M. mentalis and M. tibialis anterior) were recorded, as well as respiratory parameters and electrocardiography.

The following sleep data were analyzed: 1) Total sleep time (TST), defined as the duration of time spent in bed during a given sleep period. The calculation of this index entails the subtraction of nighttime waking phases from the total duration of sleep; 2) Sleep efficiency (SE), defined as the ratio of time spent asleep in bed to the total time spent in bed (expressed as a percentage). Concomitantly, sleep efficiency is regarded as a pivotal indicator of nighttime sleep capacity; 3) N1 stage (light sleep-transitional phase between wakefulness and sleep). This stage is characterized by a decrease in alpha waves and the emergence of theta waves; 4) N2 stage (medium sleep –distinguished by the presence of sleep spindles and K-complexes in the EEG). This stage is crucial for memory consolidation; 5) N3 stage (deep sleep – also referred to as slow-wave sleep). This phase is distinguished by the presence of delta waves, which play a pivotal role in physical recovery and immune system regulation. According to the nomenclature previously employed by Rechtschaffen and Kales, sleep phase N4 was also identified; however, since 2007, this phase has been combined with stage N3; 6) REM sleep (distinguished by rapid eye movements, nearly complete muscle atonia, and elevated brain activity). 7) Sleep onset latency (SL) (for sleep stage N1, and especially for stage N2) describes the ability to fall asleep. 8) Wakefulness after sleep onset (WASO), defined as the total time an individual is awake during the sleep period, after initially falling asleep and before waking up for the day. The presence of elevated WASO levels is indicative of suboptimal sleep quality, suggesting the occurrence of fragmented sleep. 9) Arousal index (AI) is a metric that quantifies the number of awakening responses per hour in relation to total sleep time. It serves as an indicator of sleep fragmentation. The staging of sleep was performed according to the current sleep criteria [20], [21], [22].

As a control reference, the proposed normative reference standards from a systematic review from Boulos et al. [23] were used. These values were comprised from the studies of healthy adults, male and female, aged 18–85 that reported polysomnographyic parameters using Sleep Medicine Criteria.

We also analyzed parameters associated with sleep-related breathing disorders such as the apnea-hypopnea index (AHI) and oxygen saturation levels (SpO_2_). AHI is a quantitative metric used to describe the severity of a sleep-related breathing disorder. This scale provides a quantitative measurement of apneas and hypopneas experienced during one hour of sleep. The American Academy of Sleep Medicine (AASM) provides the following classification as a guideline: AHI < 5 indicates no apnea, AHI 5–15 indicates mild sleep apnea, AHI 15–30 indicates moderate sleep apnea, and AHI ≥ 30 severe sleep apnea [24]. SpO_2_ were also monitored during the entire sleep period. Patients with healthy lungs maintain oxygen saturation levels (SpO2) between 95 and 100 percent during sleep, akin to the levels experienced while in a wakeful state. According to the AASM definition, sleep-related hypoxemia is characterized as an SpO2 of less than 88% for a duration exceeding five minutes [24].

To evaluate the potential association between sleep and disease severity in patients with dystonia, we also conducted correlation analyses between motor scores (BFMDRS and TWSTRS) and sleep parameters.

### 2.1 Statistical analysis

To compare the data of our cohort with dystonia and the normative collective, a two tailed Welch’s test was used. Descriptive statistics such as mean, standard deviation and confidence interval were obtained for all variables. A correlation analysis was performed with the Spearman Rho correlation test. The data were analyzed by SPSS software. A p value of ≤ 0.05 was considered significant.

## 3 Results

In the self-reported questionnaire PSQI patients scored 9 points (±4.3), which corresponds to impaired nocturnal sleep defining them as poor sleepers [15]. On the other hand, the mean ESS score was 4.7 (±4.5), which is considered a normal range of sleep propensity [16].

The recorded mean overnight SpO_2_ was 95.5% (±1.5), which falls within the normal physiological range. AHI measured 3.3 events per hour (±2.9), which is considered within normal limits and does not indicate presence of sleep apnea [24].

**Fig. 1:**
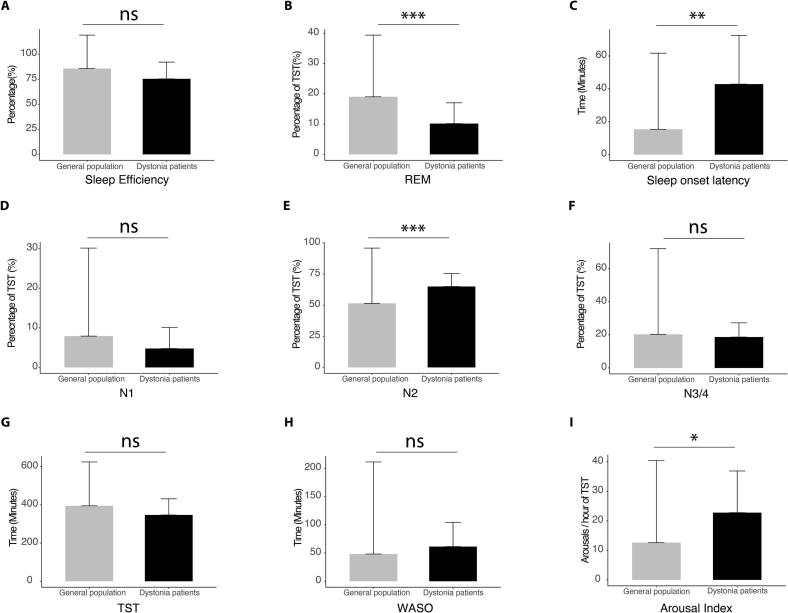
Comparison of polysomnographic parameters of patients with idiopathic dystonia with normative values (Boulos et al. 2019). A) Sleep efficiency; B) REM stage of sleep; C) Sleep onset latency, D) N1 stage of sleep; E) N2 stage of sleep; F) N3/4 stage of sleep; G) Total sleep time (TST); H) Wakefulness after sleep (WASO), I) Arousal index ***p < 0.001, **p < 0.01 *p < 0.05.

The analysis of sleep architecture showed that our patients with dystonia had a significant reduction of REM sleep duration compared to normative values [23] (p < 0.001, Fig. 1B). In contrast, the N2 sleep stage was longer in the patients with dystonia compared to normative values (p < 0.001, Fig. 1E). Finally, the patients with dystonia had prolonged sleep onset latency (p < 0.01, Fig. 1C), as well as increased AI (p < 0.05, Fig. 1I) compared to the published normative collective.

The other measured parameters such as sleep efficiency, the N1 and N3/4 phase of sleep, total sleep time and wakefulness after sleep were not significantly altered in comparison to the normative collective (Fig. 1A, 1D, 1F, 1G and 1H).

Scores for BDI and HDRS indicated mild depression (13.3 ± 5.0 and 8.2 ± 3.2, respectively). The health related quality of life scores were lower than normal in most subscales: physical functioning (p < 0.05), physical role functioning (p < 0.01), general health (p < 0.001), vitality (p < 0.05), social functioning (p < 0.01), and mental health (p < 0.05).

In order to determine whether the sleep impairments which were observed in the patients with dystonia were associated with changes in mood or quality of life, all the sleep variables, including PSQI and ESS were correlated with the BDI, HDRS and SF-36 scores. Table 1 displays the correlation matrix for these parameters showing that REM sleep is positively correlated to the SF 36-subscore mental health (Rho = 0.72, p < 0.05). Moreover, there is a significant negative association between the HDRS score and sleep stage N1 (Rho = 0.69, p < 0.05). Additionally, the arousal index was negatively related to the SF-36 subscores social functioning (Rho = -0.83, p < 0.05) and bodily pain (Rho = -0.72, p < 0.05), as well as positively correlated to the BDI depression scale (Rho = 0.63, p < 0.05). Further, patients with daytime sleepiness according to ESS had lower self-reported vitality (Rho = -0.75, p < 0.05).

**Table 1:** Correlation between sleep parameters (total sleep time-TST, sleep onset latency, Wakefulness after sleep onset-WASO, sleep efficiency, REM stage of sleep, N1 stage of sleep, N2 stage of sleep, N3/4 stage of sleep, Pittsburgh sleep quality index-PSQI and Epworth sleepiness scale-ESS) with self-reported survey SF-36 parameters (Physical functioning-PF, role physical-RF, bodily pain-BP, General health-GH, Vitality-V, social functioning-SF, role emotional-RE, mental health-MH), as well as with mood scales (Beck-Depression-Inventory-BDI and Hamilton-Depression-Rating-Scale-HDRS). Spearman Rho correlation test was used. *p < 0.05.

	**PF**		**RF**		**BP**		**GH**		**V**		**SF**		**RE**		**MH**		**BDI**		**HDS**	
	p	Rho	p	Rho	p	Rho	p	Rho	p	Rho	p	Rho	p	Rho	p	Rho	p	Rho	p	Rho
**TST(min)**	0,54	−0,04	0.97	−0.01	0.71	0.14	0.44	−0.28	0.92	0.04	0.66	0.16	0.78	0.10	0.06	0.61	0.47	−0.23	0.75	0.10
**Sleep onset latency (min)**	0,72	0,46	0.17	−0.47	0.97	−0.02	0.87	−0.06	0.66	−0.16	0.75	−0.12	0.75	0.11	0.29	−0.37	0.06	0.56	0.36	0.29
**WASO**	0,84	0,05	0.92	−0.04	0.45	−0.27	0.97	0.01	0.89	−0.05	0.33	−0.35	0.49	−0.25	0.16	−0.48	0.57	0.18	0.45	−0.24
**Sleep efficiency**	0,42	−0.27	0.44	0.28	0.45	0.27	0.60	−0.19	0.84	0.07	0.23	0.42	0.48	0.25	0.08	0.57	0.16	−0.44	0.93	0.03
**REM(% TST)**	0,22	−0,08	0.99	0.01	1.00	0.00	0.87	−0.06	0.16	0.48	0.08	0.58	0.26	0.39	**0.02**	**0.72**	0.56	−0.19	0.78	0.09
**N1(% TST)**	0,75	−0,03	0.94	0.03	0.47	0.26	0.81	−0.09	0.79	−0.10	0.52	0.23	0.29	−0.38	0.96	−0.02	0.36	−0.29	**0.01**	**−0.69**
**N2(% TST)**	0,12	−0,09	0.97	0.01	0.58	−0.20	0.77	0.10	0.41	−0.29	0.13	−0.52	0.42	−0.29	0.05	−0.63	0.83	−0.07	0.79	−0.09
**N3/4(% TST)**	0,48	0.14	0.60	0.19	0.20	0.44	0.70	0.14	0.91	0.04	0.73	0.13	0.43	0.28	0.80	0.09	0.65	0.15	0.49	0.22
**Arousal index**	0,44	0,27	0.25	−0.40	**0.02**	**−0.72**	0.25	0.48	0.33	−0.34	**0.01**	**−0.83**	0.13	−0.51	0.14	−0.50	**0.03**	**0.63**	0.69	0.13
**PSQI**	0,64	0,11	0.74	−0.12	0.45	−0.27	0.10	0.55	0.99	−0.01	0.42	−0.29	0.15	−0.50	0.42	−0.29	0.98	0.01	0.82	−0.08
**ESS**	0,13	−0,05	0.82	0.08	0.25	−0.40	0.37	−0.32	**0.01**	**−0.75**	0.52	−0.23	0.41	−0.29	0.21	−0.43	0.66	−0.15	0.28	−0.36

Correlation analyses between motor scores (BFMDRS and TWSTRS) and sleep parameters showed no significant relationship in any instance.

## 4 Discussion

Our study shows that patients with dystonia suffer from various subjective and objective characteristics of sleep impairment. While our results are overall in line with previous data, one novel aspect of our work is the correlation of sleep parameters with various domains of the quality of life scale SF-36.

Reduced quality of sleep according to the PSQI has also been reported in previous studies concentrating on patients with focal dystonia [5], [6], [12], [25]. Remarkably, however, our patients did not report daytime sleepiness according to the ESS, affirming two previous studies [6], [12], but contrasting two other studies, where ESS scores were markedly higher in dystonia patients than in control groups [26], [27]. The lack of daytime sleepiness in our cohort suggests that conventional measures of sleepiness may not capture the functional impact of sleep disruption in patients with dystonia. Therefore, sleep related impairments should be considered a multidimensional contributor to quality of life, extending beyond daytime sleep propensity alone. Nevertheless, the relatively small cohort size limited the statistical power to detect differences in daytime sleepiness in ESS.

PSG measurements revealed a significant reduction of REM sleep, increased sleep onset latency, as well as an increased arousal index, indicating sleep fragmentation. These results are in line with findings in patients with cervical dystonia [12] although in this study a reduction of sleep efficiency was reported as well. In another study no substantial difference of sleep architecture was found between patients with cervical dystonia and healthy controls [14]. In this last study, however, patients with depressive symptoms were excluded from the study. It is important to note that depression is commonly linked to sleep disturbances, such as prolonged sleep onset latency, increased REM duration and fragmented sleep [28]. In our study, patients reported only mild depression. Interestingly, the duration of the N1 stage was negatively corelated with the HDRS score. A similar correlation was observed between the arousal index and the BDI score, potentially indicating that depression might influence the sleep quality (or vice versa) in patients with dystonia.

An increased onset latency and WASO as well as reduced SE, REM sleep and TST were found in patients with generalized dystonia [29], [30]. It was thought, however, that these findings were partly attributable to medications (benzodiazepines) that half of the patients were taking. In our cohort patients were not receiving any sleep medication including benzodiazepines, indicating that sleep impairment, in particular altered sleep onset latency, increased time spent in N2 sleep, decreased time in REM sleep and an increased arousal index indeed are common primary non-motor symptoms of dystonia.

The observed differences in REM sleep, sleep onset latency, N2 sleep and arousal index are clinically relevant because they reflect alterations in sleep continuity, depth, and restorative quality, domains that are closely linked to daytime functioning and quality of life. These disturbances contribute to non-restorative sleep and have been linked with daytime fatigue and reduced cognitive performance in dystonia [5].

Surprisingly, the severity of dystonia as measured by standard rating scores (BFMDRS and TWSTRS) did not have a significant impact on sleep quality. This finding aligns with previous studies [12], [25], suggesting that the effects of the underlying disease process on sleep may be underestimated. Rather than being a secondary complication of motor symptoms, sleep disturbances may represent an independent and integral component of the disease profile as a non-motor symptom [11], [31]. The lack of significant correlation between disease severity and sleep disturbances should be interpreted in the context of the relatively low number of patients who underwent sleep assessment. In addition, disease duration did not correlate with any sleep or quality of life measures. It remains possible that studies with larger cohorts may detect more subtle associations between motor severity and sleep abnormalities, thereby further clarifying the relationship between clinical domains.

The pathogenesis of sleep disturbances in dystonia remains unclear. In dystonia abnormalities in synaptic plasticity and homeostasis have been reported in animal models and humans, suggesting that altered plasticity is a core feature of dystonia [32]. When sleep architecture is disturbed, the normal processes that stabilize synaptic connections may be impaired, which could exacerbate synaptic dysfunction and contribute to the persistence of aberrant motor patterns. Moreover, sleep disturbance has been linked to altered cerebello-thalamo-cortical connectivity and may interact with networks implicated in dystonia beyond the basal ganglia, such as the cerebellum, which itself is involved in both motor control and sleep regulation [11].

Quality of life was affected in many domains in the patients of our study, affirming previous investigations [3], [9]. The analysis of correlation between quality of life SF-36 scores and sleep parameters showed a strong correlation between the subscore mental health (MH) and the most affected sleep parameter REM, revealing a direct association of sleep disturbance and quality of life. Furthermore, a strong association was found between arousal index and the SF 36 subscales social functioning and bodily pain, suggesting that sleep fragmentation may significantly impact the social and physical dimensions of quality of life. Interestingly, although our patients did not exhibit daytime sleepiness, ESS was associated with reduced vitality, indicating that even subclinical levels of daytime sleepiness may reflect underlying sleep disturbances affecting well-being. This highlights the importance of not dismissing borderline or mildly elevated ESS scores, as they may still reflect a meaningful disruption of sleep quality, that can diminish patients’ functional status and quality of life.

Since patients in our study maintained adequate oxygenation throughout sleep and there was a lack of relevant apnea periods, our data indicate that sleep-related breathing disorders are not the cause of disturbed sleep quality in patients with dystonia.

Several limitations of our study merit consideration. Instead of comparing the PSG data to a healthy control group, we decided to use published values of a normative collective as controls. We think, however, that such an approach nevertheless allows meaningful comparisons, especially in this type of exploratory study, when establishing a control group is impractical, time-consuming and expensive. Another limitation is that the normative data were not age matched. This is an important consideration, as sleep disturbances become more prevalent with increasing age, even in healthy individuals. It is estimated that approximately 6–15% of the general population are affected with a sleep disturbance [33], which should also be taken into account when interpreting our results. The heterogeneity of our patient cohort, which comprised patients with focal, segmental and generalized dystonia, could be considered another limitation. We believe that this variability, however, enhances the generalizability of our conclusions, as all patients had idiopathic dystonia, which might introduce less bias than when including patients with defined but various genetic backgrounds and additional motor manifestations.

In conclusion, our study demonstrates that idiopathic dystonia is associated with impaired sleep quality and architecture negatively impacting mood and overall quality of life. Additionally, these results emphasize the importance of assessing sleep quality as a distinct clinical feature irrespective of motor symptom severity. Finally, our findings underscore the importance of a systematical screening for sleep-related symptoms and contributing factors, such as depression to improve patient outcomes. Comprehensive management strategies should be investigated in future studies to improve sleep quality.

## CRediT authorship contribution statement

**Lejla Paracka:** Writing – review & editing, Writing – original draft, Validation, Methodology, Investigation, Formal analysis, Data curation, Conceptualization. **Lan Ye:** Methodology, Formal analysis, Data curation. **Jann Lübke:** Software, Methodology, Formal analysis, Data curation. **Assel Saryyeva:** Resources, Data curation, Conceptualization. **Martin Klietz:** Project administration, Investigation, Data curation. **Florian Wegner:** Validation, Supervision, Methodology, Formal analysis, Conceptualization. **Joachim K. Krauss:** Writing – review & editing, Validation, Supervision, Formal analysis.

## Declaration of competing interest

The authors declare that they have no known competing financial interests or personal relationships that could have appeared to influence the work reported in this paper.
